# Dataset on sperm quality parameters and NMR-detected changes in metabolic profile of fresh and frozen turkey spermatozoa related to two different reproductive period ages

**DOI:** 10.1016/j.dib.2024.110627

**Published:** 2024-06-11

**Authors:** Giusy Rusco, Gianluca Paventi, Michele Di Iorio, Mattia Spano, Silvia Cerolini, Nicolaia Iaffaldano

**Affiliations:** a Department of Agricultural, Environmental and Food Sciences, University of Molise, 86100 Campobasso, Italy; b Department of Chemistry and Technology of Drugs, Sapienza University of Rome, 00185 Rome, Italy; c Department of Veterinary Medicine, University of Milano, 26900 Lodi, Italy

**Keywords:** Avian semen, Nuclear magnetic resonance spectroscopy, Metabolites, Cryopreservation

## Abstract

Significant changes in the quality and metabolic profile of fresh turkey sperm as a result of both cryopreservation and reproductive age have already been individually confirmed in our previous studies. This new dataset adds a relevant piece to the tangled puzzle of changes in metabolite levels affecting cryopreserved turkey sperm quality, taking into consideration two different reproductive period ages. Fresh semen samples were collected at 44 and 56 weeks of age and exposed to the cryopreservation process. All fresh and frozen-thawed samples were subjected to analysis of mobility, viability and osmotic tolerance as parameters for evaluating the sperm quality, while NMR spectroscopy was used to assess the quantitative changes in water and lipid-soluble metabolites. Our results showed that the cryopreservation process significantly affected all of the measured qualitative parameters both at 44 and 56 weeks. Concerning the metabolic profile, a greater number of quantitative changes for both water and lipid-soluble components were found in frozen semen at 56 weeks than at 44 weeks of age. These data could contribute to identifying new strategies aimed at improving freezing procedures even as reproductive age increases.

Specifications Table

**Table:** 

Subject	Biological sciences
Specific subject area	Cryopreservation of turkey semen
Data format	Analysed
Type of data	Table, Figure
Data collection	The rate of spermatozoa with a forward progressive mobility able to penetrate the Accudenz® solution, was measured by a Jasco V-570 spectrophotometer (Tokyo, Japan) at a λ of 550 nm. The results were reported as values of optical density. A fluorescence-based assay, using the stains SYBR- 14 and PI, was employed for sperm viability and osmotic tolerance analysis measured by a fluorescence microscopy (Leica Aristoplan). The results were reported as the percentage of viable spermatozoa with intact membrane. The NMR spectra were recorded with a Bruker AVANCE 600 NMR spectrometer equipped with a Bruker multinuclear z-gradient inverse probe head. The ^1^H spectra were Fourier transformed and normalized to 100.
Data source location	Department of Agricultural, Environmental and Food Sciences, University of Molise, Campobasso, Italy; Department of Chemistry and Technology of Drugs, Sapienza University of Rome, Rome, Italy.
Data accessibility	Repository name: Mendeley Data Data identification number: DOI: 10.17632/ns3x2k6c6g.1 Direct URL to data: https://data.mendeley.com/datasets/ns3x2k6c6g/1
Related research article	Paventi, G.; Di Iorio, M.; Rusco, G.; Sobolev, A.P.; Cerolini, S.; Antenucci, E.; Spano, M.; Mannina, L.; Iaffaldano, N. The Effect of Semen Cryopreservation Process on Metabolomic Profiles of Turkey Sperm as Assessed by NMR Analysis. Biology 2022, 11, 642. https://doi.org/10.3390/biology11050642

## 1 Value of the Data

• In the light of the dramatic change in sperm quality because of both cryopreservation [1] and reproductive age [2], this dataset adds a relevant piece to the tangled puzzle of changes in metabolite levels affecting sperm quality in turkey.
• Data reported in this manuscript extend that of previous studies [1,2], providing new information on the quantitative metabolite changes occurring in turkey semen cryopreservation as reproductive age increases.
• This dataset can be of interest for scientists involved in avian biology, as well as assisted reproduction and cryobiology, to better understand the biological processes involved in the cryopreservation of turkey semen related to the reproductive age, in order to identify new strategies aimed to improve freezing procedures even during the aging of male turkey.

## 2 Background

The aim of this study was to evaluate the effect of the cryopreservation process on the metabolite profile changes in turkey semen at two different reproductive period ages (44 and 56 weeks). Sperm mobility, viability and osmotic tolerance were used to evaluate the influence of freezing-thawing on semen quality parameters. The quantitative changes in water and lipid-soluble metabolites throughout semen cryopreservation were analysed by a Nuclear Magnetic Resonance (NMR) spectroscopy.

This dataset extends a previous study and adds to the pool of information, contributing to a deeper insight into the metabolite profile changes occurring in turkey sperm cryopreservation at different reproductive period ages.

## 3 Data Description

### 3.1 *Sperm quality parameters*

**Fig. 1:**
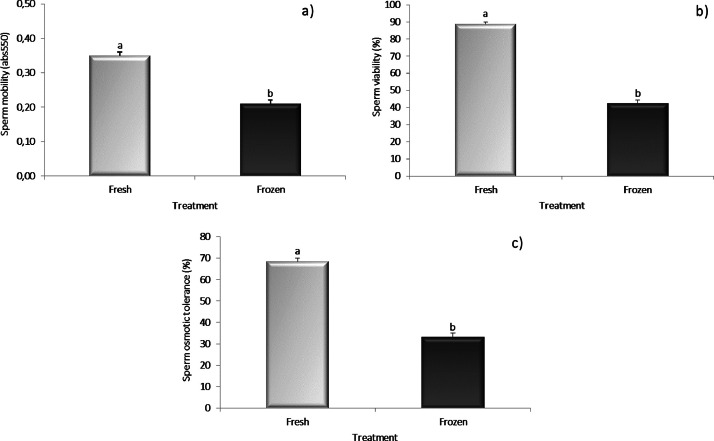
Mobility (a), viability (b) and osmotic tolerance (c) results of fresh and frozen sperm in turkey aged 44 weeks. Mean values ± SEM (*n* = 5) of sperm qualitative parameters recorded for either fresh or frozen–thawed turkey sperm were reported. The significance level was set at *p* < 0.05.

**Fig. 2:**
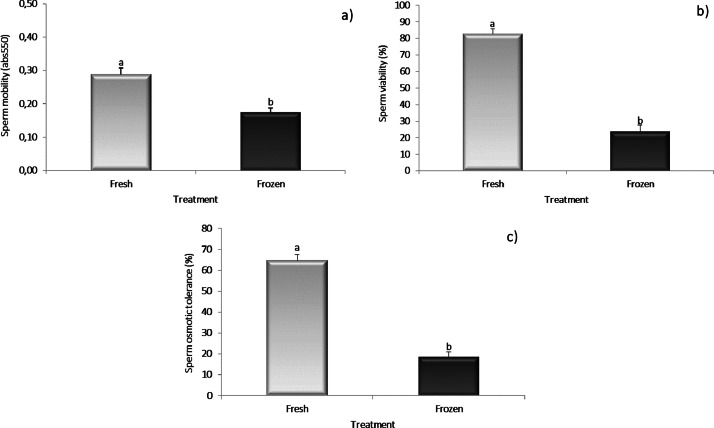
Mobility (a), viability (b) and osmotic tolerance (c) results of fresh and frozen sperm in turkey aged 56 weeks. Mean values ± SEM (*n* = 5) of sperm qualitative parameters recorded for either fresh or frozen–thawed turkey sperm were reported. The significance level was set at *p* < 0.05.

The sperm quality parameters (sperm mobility, viability and osmotic tolerance) recorded in fresh and frozen–thawed sperm samples are provided in Figs. 1 and 2. The cryopreservation process significantly affected all of the measured qualitative parameters both at 44 (Fig. 1a-c) and 56 weeks (Fig. 2a-c).

### 3.2 *NMR analysis*

In order to explore how cryopreservation affects the metabolite profiles of turkey sperm at two different reproductive period ages, namely 44 and 56 weeks, water and lipid-soluble constituents were identified in NMR spectra, following the same experimental conditions and assignments as detailed in our earlier studies [1,2]. Tables 1 and 2 present a comprehensive list of all identified and quantified metabolites for both fresh and frozen samples at 44 and 56 weeks of age, respectively.

**Table 1:** Metabolites detected through NMR analysis in the sperm of 44-week-old male turkeys, presented as means ± SEM, for both fresh and frozen samples.

Metabolite, ^1^H chemical shift (ppm)	fresh	frozen	
	**Water extract**		*p-value*
*Amino acids*	**mol% (*****n*****=5)**	**mol% (*****n*****=5)**	
Ala (1.48)	0.193 ± 0.011^a^	0.186 ± 0.019^a^	*0.814*
Asp (2.83)	0.331 ± 0.039^a^	0.247 ± 0.033^a^	*0.169*
Gln (2.45)	1.471 ± 0.121^a^	1.458 ± 0.102^a^	*0.918*
Glu (2.07)	62.180 ± 0.457^a^	65.105 ± 0.995^a^	*0.097*
Gly (3.57)	4.533 ± 0.097^a^	4.537 ± 0.094^a^	*0.958*
Ile (1.02)	0.009 ± 0.001^a^	0.014 ± 0.001^a^	*0.089*
Leu (0.96)	0.032 ± 0.003^a^	0.048 ± 0.008^a^	*0.204*
Phe (7.43)	0.015 ± 0.001^a^	0.023 ± 0.003^a^	*0.163*
Tyr (6.92)	0.023 ± 0.004^a^	0.033 ± 0.004^a^	*0.217*
Val (0.99)	0.027 ± 0.002^a^	0.038 ± 0.005^a^	*0.187*
***Organic acids***			
Acetate (1.93)	0.578 ± 0.276^a^	0.382 ± 0.155^a^	*0.622*
Citrate (2.57)	0.132 ± 0.006^b^	0.266 ± 0.022^a^	*0.005*
Formate (8.46)	0.078 ± 0.017^a^	0.061 ± 0.009^a^	*0.461*
Fumarate (6.53)	0.041 ± 0.004^a^	0.054 ± 0.007^a^	*0.237*
Lactate (1.33)	2.065 ± 0.167^a^	1.345 ± 0.080^b^	*0.028*
***Other compounds***			
Ac-carnitine (3.20)	0.119 ± 0.012^a^	0.065 ± 0.006^b^	*0.030*
AMP (8.28)	0.377 ± 0.038^a^	0.158 ± 0.014^b^	*0.005*
Carnitine (3.24)	0.111 ± 0.011^a^	0.060 ± 0.010^b^	*0.044*
Creatine (3.94)	4.336 ± 0.508^a^	2.113 ± 0.216^b^	*0.033*
Glucose (3.26 and 5.25)*	17.004 ± 0.207^a^	17.409 ± 0.808^a^	*0.623*
*Myo*-inositol (3.65)	6.346 ± 0.078^a^	6.399 ± 0.191^a^	*0.749*

	**Lipid extract**		
	**mol% (*n* = 3)**	**mol% (*n* = 3)**	

CHO (0.74)	7.451 ± 0.750^a^	5.786 ± 0.333^a^	*0.186*
SFA	29.920 ± 2.870^a^	44.161 ± 4.033^a^	*0.071*
DUFA (2.81)	3.710 ± 0.206^a^	3.517 ± 0.441^a^	*0.794*
UFA (2.08)	56.354 ± 1.948^a^	44.737 ± 4.144^a^	*0.102*
PUFA (2.86)	31.507 ± 1.073^a^	11.887 ± 4.622^b^	*0.046*
PC (3.28)	15.236 ± 1.937^b^	21.215 ± 0.964^a^	*0.031*
PE (3.21)	12.321 ± 1.040^a^	10.820 ± 1.257^a^	*0.410*
SMN (5.76)	6.274 ± 0.223^a^	5.316 ± 0.315^a^	*0.124*

a, b Different superscript letters within the same row indicate a significant difference (*P* < 0.05). *Abbreviations -* Ac-carnitine: acylcarnitine; AMP: adenosine monophosphate; CHO: cholesterol; SFA: total content of saturated fatty acids; DUFA: diunsaturated fatty acids; UFA: total content of unsaturated fatty acids; PUFA: polyunsaturated fatty acid; PC: phosphatidylcholine; PE: phosphatidylethanolamine; SMN: sphingomyelin.

**Table 2:** Metabolites detected through NMR analysis in the sperm of 56-week-old male turkeys, presented as means ± SEM, for both fresh and frozen samples.

Metabolite, ^1^H chemical shift (ppm)	fresh	frozen	
	**Water extract**		*p-value*
*Amino acids*	**mol% (*****n*****=5)**	**mol% (*****n*****=5)**	
Ala (1.48)	0.144 ± 0.009^b^	0.213 ± 0.005^a^	*0.005*
Asp (2.83)	0.324 ± 0.014^a^	0.242 ± 0.018^b^	*0.035*
Gln (2.45)	1.195 ± 0.058^a^	1.143 ± 0.073^a^	*0.683*
Glu (2.07)	63.013 ± 0.227^a^	53.723 ± 1.546^b^	*0.005*
Gly (3.57)	4.596 ± 0.060^b^	5.676 ± 0.087^a^	*<0.001*
Ile (1.02)	0.008 ± 0.000^b^	0.022 ± 0.002^a^	*0.002*
Leu (0.96)	0.026 ± 0.001^b^	0.084 ± 0.004^a^	*<0.001*
Phe (7.43)	0.012 ± 0.000^b^	0.031 ± 0.002^a^	*0.002*
Tyr (6.92)	0.020 ± 0.001^b^	0.036 ± 0.003^a^	*0.001*
Val (0.99)	0.023 ± 0.000^b^	0.053 ± 0.003^a^	*0.001*
***Organic acids***			
Acetate (1.93)	0.245± 0.776^a^	0.043 ± 0.013^a^	*0.053*
Citrate (2.57)	0.099 ± 0.012^a^	0.377 ± 0.149^a^	*0.119*
Formate (8.46)	0.051 ± 0.011^a^	0.015 ± 0.005^a^	*0.063*
Fumarate (6.53)	0.029 ± 0.002^b^	0.043 ± 0.002^a^	*0.024*
Lactate (1.33)	2.154 ± 0.151^a^	1.470 ± 0.092^b^	*0.021*
***Other compounds***			
Ac-carnitine (3.20)	0.083 ± 0.011^a^	0.086 ± 0.004^a^	*0.845*
AMP (8.28)	0.295 ± 0.014^a^	0.114 ± 0.008^b^	*<0.001*
Carnitine (3.24)	0.104 ± 0.009^a^	0.093 ± 0.001^a^	*0.265*
Creatine (3.94)	3.834 ± 0.282^a^	2.421 ± 0.092^b^	*0.003*
Glucose (3.26 and 5.25)*	17.325 ± 0.233^b^	25.334 ± 1.453^a^	*0.005*
*Myo*-inositol (3.65)	6.416 ± 0.101^b^	8.781 ± 0.151^a^	*<0.001*

	**Lipid extract**		
	**mol% (*n* = 3)**	**mol% (*n* = 3)**	

CHO (0.74)	8.292 ± 0.697^a^	5.631 ± 0.448^a^	*0.114*
SFA	39.489 ± 1.963^b^	55.753 ± 1.337^a^	*0.011*
DUFA (2.81)	4.873 ± 0.115^a^	2.055 ± 0.454^b^	*0.016*
UFA (2.08)	47.585 ± 1.242^a^	32.955 ± 1.620^b^	*0.017*
PUFA (2.86)	20.969 ± 1.228^a^	6.765 ± 1.593^b^	*0.037*
PC (3.28)	17.987 ± 0.093^b^	23.630 ± 0.839^a^	*0.017*
PE (3.21)	10.786 ± 0.369^a^	10.592 ± 0.233^a^	*0.720*
SMN (5.76)	4.635 ± 0.832^a^	5.662 ± 0.208^a^	*0.355*

a, b Different superscript letters within the same row indicate a significant difference (*P* < 0.05). *Abbreviations -* Ac-carnitine: acylcarnitine; AMP: adenosine monophosphate; CHO: cholesterol; SFA: total content of saturated fatty acids; DUFA: diunsaturated fatty acids; UFA: total content of unsaturated fatty acids; PUFA: polyunsaturated fatty acid; PC: phosphatidylcholine; PE: phosphatidylethanolamine; SMN: sphingomyelin.

The identified water-soluble metabolites included different classes of compounds, such as amino acids, organic acids and other constituents. At 44 weeks, no significant differences between fresh and frozen–thawed samples were recorded for amino acids; in terms of organic acids and other compounds, frozen–thawed semen exhibited a reduction in the content of lactate, Ac-carnitine, AMP, carnitine and creatine compared to the fresh semen (*p* < 0.05), whereas citrate levels were significantly increased in cryopreserved samples. Concerning the lipid extract, a significantly higher value was recorded in fresh sperm than in frozen–thawed sperm only in polyunsaturated fatty acids (PUFA), whereas the levels of phosphatidylcholine (PC) showed an increase in the cryopreserved samples (*p* < 0.05).

With respect to what was observed at 44 weeks, a greater number of quantitative changes for both water and lipid-soluble components were found at 56 weeks (Table 2). In particular, all levels of amino acids identified underwent a significant change due to the cryopreservation process, except for glutamine (Gln); the amount of aspartic acid (Asp) and glutamate (Glu) decreased in frozen-thawed semen (*p* < 0.05), while alanine (Ala), glycine (Gly), leucine (leu), isoleucine (Ile), phenylalanine (Phe) tyrosine (Tyr) and valine (Val) levels showed an opposite trend after cryopreservation. Among organic acids and other compounds, frozen-thawed semen showed notable decreases in lactate, AMP and creatine (*p* < 0.05), conversely, fumarate, glucose and myo-inositol values were increased (*p* < 0.05). There were no significant differences between fresh and frozen semen when it came to acetate, citrate, formate, Ac- carnitine and carnitine.

In lipid-soluble metabolites, significantly higher values of unsaturated fatty acids (DUFA), (UFA) and PUFA were observed in fresh than in frozen–thawed sperm, whereas higher levels of saturated fatty acids (SFA) and PC were found in frozen-thawed samples (*p* < 0.05). There were no significant differences between fresh and frozen–thawed samples in the other identified lipids.

## 4 Experimental Design, Materials and Methods

### 4.1 *Animals and semen cryopreservation procedure*

For the experiment design Hybrid Large White turkey males raised under standard management conditions by a private breeding group (Agricola Santo Stefano of Amadori's group, Canzano, TE, Italy) were used. Animals were exposed daily to a photoperiod of 14L:10D, kept in groups of 8–10 in floor pens and fed and watered *ad libitum*.

Semen samples were collected at 44 and 56 weeks of age following a previous period of training the turkeys through abdominal massage (twice a week). Five distinct semen pools (4 mL of semen/ pool) were obtained from a range of 9–12 males for each reproductive age, to avoid the effects of individual differences among males. Each pool was divided into two aliquots, one to be immediately assessed (fresh semen) as described below, and the other to be subjected to the freezing–thawing process.

Semen cryopreservation was conducted according to the pellet method as described in Iaffaldano et al. [3]. In brief, each semen pool was initially diluted with Tselutin extender in a 1:4 vol ration (v/v) [4]. Subsequently, samples were cooled at 4 °C for one hour, after which 8 % (v/v; 0.860 M) of dimethylacetamide (DMA) was added as cryoprotectant. The semen was gently mixed and equilibrated at 4 °C for 5 min. Semen (0.080 mL total volume) was plunged drop by drop directly into liquid nitrogen (LN_2_) to form spheres of frozen semen (pellets) and then rapidly placed in 2 mL polypropylene cryovials (Cryo.sTM; Greiner Bio-one, Monroe, NC, USA), previously cooled by immersion in LN_2_ (3–4 pellets/cryovial). Samples were stored in a liquid nitrogen tank for two weeks, after which they were subjected to analysis followed by thawing of the cryovials in a water bath at 75 °C for 12 s.

### 4.2 *Semen quality evaluation*

All sperm quality parameters (mobility, viability and osmotic tolerance) were evaluated on fresh and cryopreserved samples at 44 and 56 weeks of age.

Sperm mobility was analysed by spectrophotometric measurement of the spermatozoa rate with a forward progressive mobility able to penetrate a biologically inert cell-separation solution consisting of Accudenz® (Accurate Chemical & Scientific Corp., Westbury, NY 11590). Fresh and cryopreserved semen samples were diluted to 1.0 × 10^9^ sperm/mL in a solution consisting of 111 mM NaCl buffered with 50 mM N-tris-[hydroxymethyl]methyl-2 amino-ethanesulfonic acid (TES), pH 7.4, containing 25 mM glucose and 4 mM CaCl_2_ [2]. An aliquot of each sperm suspension (0.060 mL) was carefully overlaid on 0.600 mL of pre-warmed Accudenz® solution at a 4 % concentration (w/v) [5], in a semimicro polystyrene disposable cuvette. Subsequently, they were incubated in a water bath at 41 °C for 5 min and placed in a spectrophotometer (Jasco V-570, Tokyo, Japan), where the absorbance was measured at a λ of 550 nm after 1 min. The values of optical density (O.D.) were used to measured sperm mobility.

Sperm viability evaluation was performed by a LIVE/DEAD sperm viability kit (Molecular Probe, Eugene, OR, USA), which is based on a dual staining technique, using SYBR- 14 and Propidium Iodide (PI). SYBR-14 is a membrane-permeant DNA probe which is able to selective stain only the nuclei of viable sperm, emitting a fluorescent bright green color. PI does not incorporate into live sperm with intact membranes; therefore, it is able to detect only non-viable spermatozoa by emitting a fluorescent red color.

The analysis was performed as described by Iaffaldano et al. [3]. Specifically, the first step was to dilute the SYBR-14 with dimethylsulfoxide (DMSO) in a 1:100 vol ratio (v:v). Subsequently, volumes of 5 µL diluted semen were added to 0.039 mL of Tselutin containing 1 µL of diluted SYBR. Samples were incubated at 37 °C for 10 min, after which 5 µL of PI (previously dissolved 1:100 in PBS) were added to samples that were further incubated at 37 °C for 5 min. Viable/non-viable spermatozoa were evaluated by a fluorescence microscopy (Leica Aristoplan; Leitz Wetzlar, Heidelberg, Germany), using a blue excitation filter at a λ of 488 nm, ×100 oil immersion objective and magnification × 400. Approximately 200 spermatozoa were counted and the percentages of viable spermatozoa were calculated as follows:

%ofspermviability=greencells/(greencells+redcells)×100

The hypo-osmotic swelling test (HOST) was used to evaluate the percentage of sperm osmotic tolerance *in vitro*. The principle of which this is based is that the viable cells exposed to a hypo-osmotic environment allows the passage of water into the cytoplasmatic space causing swelling without damaging the membrane; when the membrane remains intact only the SYBR- 14 can penetrate it, emitting a fluorescent green color. Conversely, sperm with damaged membrane allows PI to enter, resulting in the red staining of sperm cells for which the functional integrity was compromised.

In agreement with our previous study [6], aliquots of 5 µL of semen were added to 0.080 mL of distilled water, stained with SYBR-14 and PI and then read as described above for sperm viability analysis.

### 4.3 *NMR measurements*

#### 4.3.1 Sample preparation

For the NMR analysis, fresh and frozen semen samples were adjusted to the same concentration (15 × 10^9^ spermatozoa) and subjected to centrifugation at 1500 rpm for 15 min to eliminate the diluent and seminal plasma. The Bligh-Dyer protocol [7], was employed to extract both polar and apolar metabolites from semen samples (pellets), following the procedure described by Ingallina et al. [8]. In this regard, a chloroform/methanol solution in a 2:1 vol ratio (v/v) was added to the pellets and the obtained system was vigorously homogenized for one minute using a vortex mixer. Distilled water was then added in a ratio of 1:18:4 (spermatozoa–chloroform/methanol–water). The obtained homogenate was subjected to centrifugation at 4000 rpm for 20 min at a temperature of 5 °C, leading to the separation of liquid organic and hydroalcoholic phases, that were subsequently dehydrated under a vacuum using a rotary evaporator. The dried extracts were then reconstituted in either 0.75 mL of CDCl_3_/CD_3_OD (2:3, v/v) or 0.75 mL of D_2_O phosphate buffer (400 mM, pD = 7).

#### 4.3.2 Acquisition of the NMR spectra

NMR spectra for the aqueous and organic extracts were acquired at a temperature of 27 °C using a Bruker AVANCE 600 NMR spectrometer, which operated at a proton frequency of 600.13 MHz. This spectrometer was equipped with a Bruker multinuclear z-gradient inverse probe head capable of generating z-direction gradients with a strength of 55 G/cm. ^1^H spectra were referenced to methyl group signals of 3 (trimethylsilyl)-propionic-2,2,3,3-d4 acid sodium salt (TSP, δ= 0.00 ppm) in D_2_O and to the residual CHD_2_ signal of methanol (set to 3.31 ppm) in CDCl_3_/CD_3_OD mixture [9]. For ^1^H spectra acquisition of aqueous extracts, a total of 512 transients were used, with a 3-second recycle delay. Bruker presaturation sequence zgpr was employed to suppress the residual HDO signal. Experiments were carried out using a 45° pulse of 7.25 µs and 32 K data points. ^1^H spectra of organic extract were acquired using 256 transients, 32 K data points, a 3-second recycle delay, and a 90° pulse lasting 10 µs. All acquired ^1^H spectra underwent Fourier transformation with an exponential multiplication function featuring a line broadening factor of 0.3 Hz and a manual phase correction and baseline correction were applied. This processing procedure was performed using Bruker TOPSPIN software, version 1.3.

#### 4.3.3 Measurement of the metabolomic content in aqueous extract

The intensity of 21 ^1^H resonances associated with polar metabolites, as specified in Table 1 and 2, was measured by comparing them to the TSP signal area, normalized to 100, and used as an internal reference standard.

#### 4.3.4 Measurement of the metabolomic content in organic extracts

The integrals of 8 ^1^H resonances corresponding to assigned lipo-soluble metabolites were measured and used to obtain the normalized integrals, see Table 1 and 2. In particular, signal areas were normalized with respect to those of α-CH_2_ groups found in all fatty acid chains at 2.31 ppm, which were set to 100 %. The molar percentages of lipids were then determined, considering the number of equivalent protons for each used signal. The resonances considered for integration included: CH_3_ of cholesterol (0.74 ppm), allylic protons of all UFA (2.08 ppm), α -CH_2_ groups from all fatty acids (2.31 ppm), CH_2_ diallylic protons of DUFA (2.81 ppm), CH_2_ diallylic protons of PUFA (2.88 ppm), CH_2_N of PE (3.21 ppm), (CH_3_)_3_N^+^ of PC (3.28 ppm), and CH (double bond) proton of SMN (5.76 ppm).

### 4.4 *Statistical analysis*

Sperm qualitative parameters and metabolite levels determined by NMR analysis measured in fresh and frozen–thawed sperm for both reproductive ages (44 and 56 weeks) were compared by a paired-samples *t*-test (threshold at *p* < 0.05). Statistical tests were performed using the software package SPSS v23.0 (SPSS, Chicago, IL, USA).

## Limitations

Not applicable.

## Ethics Statement

The handling of animals and semen collection was conducted in accordance with the Code of Ethics of the EU Directive 2010/63/EU.

## CRediT authorship contribution statement

**Giusy Rusco:** Investigation, Writing – original draft, Writing – review & editing. **Gianluca Paventi:** Conceptualization, Methodology, Formal analysis, Data curation, Writing – review & editing, Visualization. **Michele Di Iorio:** Conceptualization, Methodology, Formal analysis, Investigation, Data curation, Writing – review & editing, Visualization. **Mattia Spano:** Investigation. **Silvia Cerolini:** Validation, Writing – review & editing. **Nicolaia Iaffaldano:** Conceptualization, Methodology, Validation, Formal analysis, Writing – review & editing, Supervision, Funding acquisition.

## Data Availability

Raw data - semen quality (44 & 56 weeks); Raw data from NMR (44 & 56 weeks) (Original data) (Mendeley Data)
